# Preface

**DOI:** 10.1186/s12859-019-2765-x

**Published:** 2019-06-10

**Authors:** Henry Han, Wenbin Liu

**Affiliations:** 1 Department of Computer and Information Science, Fordham University, Lincoln Center, 113 W. 60th Street, New York, NY 10023 USA; 2 Institute of Computational Science and Technology, Guangzhou University, College city 240, Guangzhou, 510006 China

Recent surges in bioinformatics, medicine, and data science are changing the world broadly and deeply at an unprecedented speed. It not only brings a sheer amount of new data (e.g., scRNA-seq data) and challenges, but also the new opportunities, technologies, and breakthroughs in the exciting interdisciplinary fields. The International conference on Data science, Medicine and Bioinformatics (IDMB) provides an international forum for the presentation of original research results in the new interdisciplinary fields, as well as dissemination and exchange of novel data handling and methodology experiences.

This IDMB 2018 BMC bioinformatics special issue covers some most challenging and exciting state-of-the-art investigations in bioinformatics and data science, besides interesting topics in translational  research. For examples, our investigators employ state-of-the-art machine learning strategies to identify antimicrobial peptides and their function types for penaeus, which has been a long time viewed as a topic in marine biology technically. Moreover, a new probe computing model based on the small molecular switch is proposed by a research team consisting of computer scientists and biologists, besides developing a novel rigorous graph regularized matrix factorization method to predict drug-target interactions. In addition, a data science approach is developed to predict the pandemic risk of avian influenza viruses via scoring amino acid mutations, which is an interaction between virology and bioinformatics. Such a study represents an important new direction in the two sibling fields.

Furthermore, there are two papers included in this special issue on using novel evolutionary computing algorithms: enhanced fruit fly optimization and new particle swarm optimization to find good features and gene marker prediction in complex disease diagnosis. They present novel AI techniques in bioinformatics and medicine fields and will continue to grow to the next level with the surge of artificial intelligence techniques in health and medicine fields.

Single cell transcriptomics is becoming an important field in bioinformatics and medicine by making it possible to investigate transcriptomic landscapes at the single-cell resolution. However, it is still unclear about how to conduct effective phenotype clustering for  single-cell RNA-seq data. A novel research effort by combining gene ontology with deep neural networks to tackle this issue is proposed in an interesting paper in this special issue. It will represent the research direction to tackle large scale omics data analysis problem via deep learning. Finally, an interesting paper to develop new entropy techniques to detect exon and intron in genomics is also presented in this special issue. It may inspire more classic sequence analysis research with a novel data science technique.

We thank all colleagues to make a successful IDMB 2018 and look for more exciting IDMB2019!

